# Next‐generation sequencing in identification of pathogenic variants in primary hyperoxaluria among 21 Egyptian families: Identification of two novel *AGXT* gene mutations

**DOI:** 10.1002/mgg3.1992

**Published:** 2022-06-03

**Authors:** Hoda A. Ahmed, Fatina I. Fadel, Mohamed A. Abdel Mawla, Doaa M. Salah, Mohamed Gamal Fathallah, Khalda Amr

**Affiliations:** ^1^ Medical Molecular Genetics Department, Human Genetics and Genome Research Division National Research Centre Cairo Egypt; ^2^ Pediatrics Department, Faculty of Medicine Cairo University Giza Egypt; ^3^ Pediatrics Department National Research Centre Cairo Egypt

**Keywords:** *AGXT*, mutations, NGS, PH, recessive

## Abstract

**Background:**

Primary hyperoxaluria (PH) is a rare heterogeneous, autosomal recessive disorder of glyoxylate metabolism. It is characterized by excessive hepatic production of oxalate resulting in a wide spectrum of clinical, imaging, and functional presentation. The characteristic features of PH comprise of recurrent urolithiasis, renal stones, and/or nephrocalcinosis. Three known types of PH have been identified PH1, PH2, and PH3. Pathogenic variants in *AGXT*, *GRHPR*, and *HOGA1* cause the phenotypic expression of PH.

**Methods:**

In this study, we describe the clinical and genetic findings of 22 patients from 21 unrelated Egyptian families with the distinctive clinical features of PH. A thorough clinical evaluation followed by an NGS custom panel of *AGXT*, *GRHPR*, and *HOGA1* genes was done.

**Results:**

Two novel mutations (p.Gly27Glu and p.Gln256Serfs*17) and six previously reported mutations (p.Lys12Glnfs*156, p.Lys12Argfs*34, p.Ile244Thr, p.Asn22Ser, p.Pro11Leu, and p.Ile340Met) were identified in *AGXT* gene. The NGS panel results were validated thereafter using Sanger sequencing.

**Conclusion:**

Our results extend the number of *AGXT* mutations identified so far and emphasize the important role of genetic testing in providing proper counseling and patients management.

## Graphical abstract

Primary hyperoxaluria (PH) is a rare heterogeneous, autosomal recessive disorder of glyoxylate metabolism. It is characterized by excessive hepatic production of oxalate resulting in wide spectrum of clinical, imaging and functional presentation. The characteristic features of PH comprise of recurrent urolithiasis, renal stones and/or nephrocalcinosis. Three known types of PH have been identified PH1, PH2 and PH3. Pathogenic variants in AGXT, GRHPR and HOGA1 cause the phenotypic expression of PH.

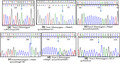

## 1 INTRODUCTION

Primary hyperoxaluria (PH) is a genetically heterogeneous disorder. Three types of PH have been identified that are inherited in an autosomal recessive pattern including PH1 (OMIM; 259,900), PH2 (OMIM; 260,000), and PH3 (OMIM; 613,616). The main features of the disease include excessive hepatic production of oxalate leading to recurrent nephrolithiasis, crystalluria, nephrocalcinosis, and end‐stage kidney disease (ESKD). Calcium oxalate crystals also can deposit in various organs like the retina, heart, blood vessels, bones, and other organs (oxalosis) leading to severe morbidity that may cause death (He et al., 2019; Soliman et al., 2017).

Primary hyperoxaluria type 1 (PH1) is the most common and devastating subtype accounting for about 80% of all patients. It is caused by deficiency of the liver‐specific, peroxisomal enzyme alanine‐glyoxylate aminotransferase (AGT) which is encoded by *AGXT* gene. On the other hand, PH2 is less severe than PH1 and caused by a deficiency of glyoxylate and hydroxypyruvate reductase, which is encoded by the *GRHPR* gene. The deficiency of the mitochondrial enzyme, 4‐hydroxy‐2‐oxoglutarate aldolase (HOGA1), is responsible for PH3 which is representing the least severe type. The clinical manifestation of the three types of PH is very similar including recurrent nephrolithiasis and progressive nephrocalcinosis with renal stones. The age of onset is also similar, and management varies according to the type of PH so an early and accurate diagnosis is essential to guide appropriate treatment. Increased excretion of urinary oxalate is seen in all forms of PH although neither urine nor plasma oxalate is diagnostic because it is elevated in other causes of renal failure (He et al., 2019; Rumsby, 2015).

Diagnosis of PH depends on many diagnostic tools including biochemical analysis of urine, the stone composition of pure calcium oxalate monohydrate, and genetic analysis (Soliman et al., 2017). A definitive diagnosis of PH currently requires a measurement of enzyme catalytic activity by an invasive approach of needle liver biopsy with special strict conditions for sample transport (Kanoun et al., 2017). Molecular analysis of PH is an efficient, noninvasive alternative approach to confirm the diagnosis of PH. Herein, we describe the clinical and molecular findings of 22 Egyptian patients with PH by using NGS analysis to identify the causative variants among these patients as a step toward understanding the pathogenesis of PH. This study is the first molecular study in Egypt describing the mutations causing PH.

## 2 SUBJECTS AND METHODS

### 2.1 Editorial policies and ethical considerations

This study was approved by the Ethical Scientific Committee of the National Research Centre (NRC), Cairo, Egypt and conducted in accordance with the declaration of Helsinki ethical principles for medical research involving human subjects. An informed consent was obtained from the patients or their guardians.

### 2.2 Patients

The study included 22 patients descending from 21 Egyptian unrelated families. Patients were recruited from the outpatient clinics of the Pediatric Nephrology Unit, Cairo University. Their age at presentation ranged from 11 months to 6 years. All patients were subjected to full medical history taking, three generations pedigree construction, and clinical examination. Urine oxalate/creatinine ratios, plasma oxalate, and abdominal ultrasonography were performed in all studied patients. Bone X‐rays and echocardiography were conducted on some patients. Measurement of AGT enzyme activity was conducted in some patients. Secondary causes of hyperoxaluria such as intestinal malabsorption and inflammatory bowel disease were excluded.

### 2.3 Targeted next‐generation sequencing (NGS)

Genomic DNA was extracted using the slandered method from peripheral blood lymphocytes of patients and their parents. A customized NGS panel was designed and the targeted genomic regions included the exons, around 100 bp of flanking intronic sequences, and the 5′ and 3′ untranslated regions of the three primary hyperoxalurias causing genes *AGXT* (NM_000030.3), *GRHPR* (NM_012203.2), and *HOGA1* (NM_138413.4). The average coverage depth of the entire panel was 100× and ~85%–100% of targeted bases were covered. The NGS library was prepared using the TruSeq Custom Amplicon kit (Illumina). Briefly, 500 ng of gDNA in 5 μl water were hybridized with an oligo pool. Then, unbound oligos were removed and extension‐ligation of bound oligos was followed by PCR amplification. PCR products were cleaned and checked for quality using Tapestation analysis (Agilent). Prior to sequencing, libraries were normalized with the normalization process of the Truseq Custom Amplicon kit. Libraries were paired‐end sequenced with 2*151 bp cycles on a MiSeq device (Illumina). Sequence data were aligned to the GRCh37/hg19 reference sequence, and data analysis was carried out using IlluminaVariantStudio software. Sequence data were visualized using the Integrative Genomics Viewer (IGV, http://www.broadinstitute.org/igv/). All variants with satisfying sequencing depth and quality were filtered according to their frequency in dbSNP 141, 1000G, and gnomAD exome (with MAF ≤ 0.01). (http://gnomad.broadinstitute.org/). Previously reported disease‐causing variants were also considered. Variants were classified according to the ACMG‐AMP guidelines.

### 2.4 Sanger sequencing for validation of NGS data

All identified variants were confirmed by Sanger sequencing and validated by parental testing. Exons encompassing the *AGXT* (NM_000030.3) variants were amplified using specific primers designed by ExonPrimer Software. PCR products were purified using the Exo‐SAP PCR Clean‐up kit (Table 1).

**TABLE 1:** Clinical manifestations of the studied Egyptian primary hyperoxaluria (PH) patients

Family	Patient	Sex	Age of onset	Consanguinity	Family history	Nephrolithiasis	Nephrocalcinosis	Infection	ESRD	Hydronephrosis	On dialysis	Systemic oxalosis	Oxaluria μmol/24 h)
F1	P1	M	11 m	+	+	+	+	+	+	+	Yes	Myocardial infiltrations and left ventricular hypertrophy Bone deformities	821 μmol/24 h
F2	P2	M	1 y	+	+	+	−	−	+	+	Yes	Bone deformities	750 μmol/24 h
F3	P3	M	6 m	+	−	+	+	+	+	−	Yes	Bone deformities	876 μmol/24 h
F4	P4	F	10 m	−	−	+	−	−	+	−	Yes	Bone deformities Retinal deposition of oxalates and unilateral loss of vision	605 μmol/24 h
F5	P5	M	1 y 2 m	+	+	+	+	+	+	+	Yes	Myocardial infiltrations and left ventricular hypertrophy	798 μmol/24 h
F6	P6	M	1 y 5 m	−	+	+	−	−	+	−	Yes	Bone deformities	630 μmol/24 h
F7	P7	F	11 m	+	−	+	+	+	+	+	Yes	Myocardial infiltrations and left ventricular hypertrophy Bone deformities	781 μmol/24 h
F8	P8	F	8 m	+	−	+	+	−	+	−	Yes	Bone deformities	805 μmol/24 h
F9	P9	M	9 m	+	+	−	+	+	+	+	Yes	Myocardial infiltrations and left ventricular hypertrophy	642 μmol/24 h
F10	P10	M	1 y 6 m	+	+	+	−	+	+	+	Yes	−	621 μmol/24 h
F11	P11	F	5 y 6 m	+	+	−	+	+	−	−	No	Bone deformities	532 μmol/24 h
F12	P12	M	1 y 4 m	+	−	+	+	+	+	+	Yes	Bone deformities	760 μmol/24 h
F13	P13	M	2 y	+	+	+	+	_	+	−	Yes	Bone deformities	540 μmol/24 h
F14	P14	F	1 y 11 m	+	+	+	+	−	+	−	Yes	Bone deformities	679 μmol/24 h
F15	P15	F	2 y 3 m	+	−	−	+	+	+	−	yes	Bone deformities	549 μmol/24 h
F16	P16	M	2 y 7 m	−	−	+	−		+		Yes	Retinal deposition of oxalates and unilateral loss of vision	767 μmol/24 h
F17	P17	F	1 y 2 m	+	+	+	−	−	+	+	Yes	Myocardial infiltrations and left ventricular hypertrophy	760 μmol/24 h
F18	P18	M	2 y 2 m	+	+	+	−		+	+	yes	−	578 μmol/24 h
F19	P19	M	6 y 7 m	+	+	+	+	−	−	−	No	−	476 μmol/24 h
F19	P20	M	5 y 1 m	+	+	+	+	−	−	−	No	−	567 μmol/24 h
F20	P21	M	11 m	−	−	+	+	−	+	−	Yes	Bone deformities	746 μmol/24 h
F21	P22	M	6 y	−	−	−	+	−	−	−	No	−	465 μmol/24 h

### 2.5 Prediction of the potential pathogenicity of the novel variants

To evaluate the deleterious effect of novel variants, in silico analysis was used (Table 2). Moreover, to evaluate the mutation effect on protein, 3D structure Swiss‐Pdb Viewer version 4.1.0 was used to elucidate the crystallographic structure of the human Alanine‐glyoxylate aminotransferase (AGXT) at a resolution of 2.5 Å (PDB: 1h0c). And then the mutation effect was analyzed using Single Amino Acid Mutation change of Binding Energy (SAAMBE), Missense 3D, and PremPS tools to evaluate the effect of a single mutation on protein stability by calculating the quantitative changes in unfolding Gibbs free energy (Figure 2).

**TABLE 2:** Prediction of the potential pathogenicity of the novel variants

Name of the variants	gnomAD MAF	ExAC MAF	1000 Genome	Mutation taster prediction and score	PolyPhen‐2.0 prediction and score	SIFT	PhD‐SNP	Mutation Assessor	PROVEAN	SNPs &GO	MutPred	PROVEAN
c.80G > A p.Gly27Glu	N/A	N/A	N/A	Disease causing (prob:0.99)	Probably damaging (1.000)	Damaging (0.001)	Disease (RI = 7)	High (4.05)	Deleterious (−7.27)	Disease (RI = 10)	Deleterious (0.886) Altered Transmembrane protein (0.9; 0.05) Loss of N‐terminal acetylation at M1 (0.02; 7.0e‐03)	Deleterious (−7.27)
c.766delC p.Gln256Serfs*17	N/A	N/A	N/A	disease causing (prob: 1)	–	–	–	–	–	–	–	–

*Notes*: PHD‐SNP & SNPs & GO: directly predicts disease effect and gives Reliability Index range from 0 to 10. 0 unreliable and 10 reliable; MutationAssessor: predicted functional, that is, high (“H”) or medium (“M”) or predicted nonfunctional, that is, low (“L”) or neutral (“N”). Score cutoffs between “H” and “M,” “M” and “L,” and “L” and “N,” are 3.5, 1.935, and 0.8, respectively; PROVEAN: scores ≤−2.5 is predicted as “Damaging”; otherwise, it is predicted as “Neutral”; REVEL, PolyPhen2, and MutPred: score ranges from 0.0 (tolerated) to 1.0 (deleterious); SIFT: threshold for intolerance is 0.0.

## 3 RESULTS

### 3.1 Clinical and biochemical data

The study included 22 patients with PH; most of the patients (77%) were the offspring of consanguineous families (17/22). Patients were 15 males (68%) and 7 females (32%). Family history of similar conditions was evident in 13 independent pedigrees. All patients in the current study exhibited the characteristic clinical and radiological manifestations of PH1 with variable age of onset. Thirty‐two percent (7/22) of studied patients presented during their first year of life, 50% (11/22) of patients presented prior to the age of 5 years, and the remaining 18% (4/22) patients had their first symptoms after the age of 5 years. Nephrolithiasis was noted in (18/22) and nephrocalcinosis on (9/22). Progressive ESKD was recorded on 18/22. Elevated plasma and urinary oxalate levels were detected in all patients. Renal ultrasonography revealed multiple renal stones in 18/22 of whom 9 patients had the complication of nephrolithiasis (infection and obstruction). The extrarenal manifestation was reported in 15 patients of whom 13 patients have bone deformities. Myocardial infiltrations and left ventricular hypertrophy were noted in five patients. Retinal deposition of oxalate and unilateral loss of vision was observed in two patients.

### 3.2 Molecular and bioinformatic findings

**FIGURE 1:**
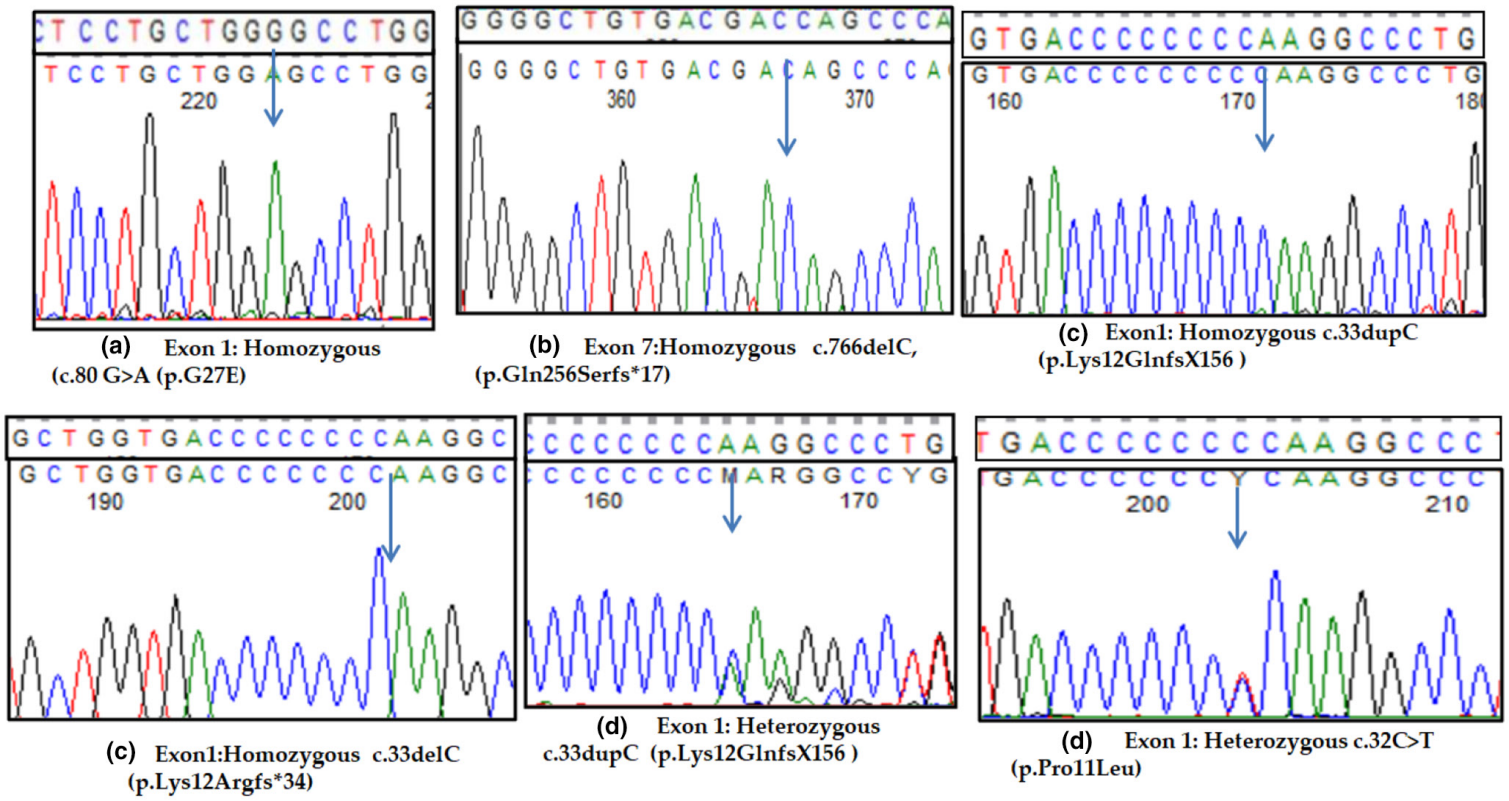
Sequencing chromatograms of *AGXT* mutations identified in our cohort. (a–b) Two novel *AGXT* mutations. (c–d) Previously reported *AGXT* mutations. Wild‐type sequences are shown above each chromatogram and arrows point toward single base changes.

A total of eight different pathogenic variants were detected in the 22 patients in *AGXT* gene NG_008005.1 (Figure 1). Two of these variants were novel: c.80G > A (p.Gly27Glu) and c.766del (p.Gln256Serfs*17) and each of them was identified in one family in a homozygous state (Figure 1a,b). The first new missense mutation c.80G > A (p.Gly27Glu) was found in two patients (family 19) proving its cosegregation with the disease phenotype. The remaining six identified mutations were previously reported c.33dupC (p.Lys12Glnfs*156), c.33delC (p.Lys12Argfs*34), c.32C > T (p.Pro11Leu), c.65A > G (p.Asn22Ser), c.731 T > C (p.Ile244Thr), and c.1020A > G (p.Ile340Met) and disclosed in 19 patients. The reported insertion mutation in exon1 c.3dupC (p.Lys12Glnfs*156) was shown the most frequent mutation among our cohort 11/22 (50%) (1‐11F) in either homozygous or the heterozygous state (Figure 1c,d). The second most common detected mutation was c.33delC (p.Lys12Argfs*34) in 6/22 (12–18F), all in a homozygous state (Figure 1c). Four families 4/22 (8–11 F) carrying compound heterozygous‐reported variants were c.33dupC (p.Lys12Glnfs*156) and c.731T>C (p.Ile244Thr) (Figure 1d). Also, c.65A>G (p.Asn22Ser) missense variant was found in a compound heterozygous with minor allele c.1020A>G (p.Ile340Met) in only one patient (P22). Nevertheless, the coinheritance of both minor alleles c.32C>T (p.Pro11Leu) and c.1020A>G (p.Ile340Met) among four studied patients were detected. The two novel identified mutations were not reported before in the dbSNP, 1000G, and gnomAD, and they were not detected in 100 normal chromosomes of Egyptian origin by Sanger sequencing and were predicted to be disease caused by in silico analysis (Table 3). According to ACGM‐AMP guidelines of variants interpretation, this variant c.766del (p.Gln256Serfs*17) causes an unambiguous pathogenic effect on the AGXT protein, and c.80G>A (p.Gly27Glu) was classified as a pathogenic variant (class5) (Richards et al., 2015). In addition, eight nonpathogenic variants were detected, seven of these variants were intronic: g.9405G>T(rs12997245), g.12312G>A(rs12695032), g.5665GGCCTCCCT(rs180177209), g.10335C>T (rs11693280), g.14856G>A (rs35566646), g.15118C>A, (rs4273214), and g.7746G > A (no rs) and one exonic variant NM_000030.3:c.132G > A (p.Gln44=).

**TABLE 3:** *AGXT* (NM_000030.3) variations detected in the studied patients with PH1

Fam	Patient	Exon	Mutation	Type of mutation	Genotype	Reference^c^
			Nucleotide change^a^			
			Protein changes^b^			
F1‐7	P1‐7	E1	c.33dupC p.Lys12Glnfs*156	Frameshift/insertion	Homozygous	Pirulli et al. (1999)
F8‐11	P8‐11	E1,7,10	c.[33dupC];[32C > T;731 T > C;1020A > G] p.[Lys12Glnfs*156]; [Pro11Leu;Ile244Thr;Ile340Met]	Frameshift/Insertion Missense	Compound Heterozygous	Purdue et al. (1990), Von Schnakenburg and Rumsby (1997), Pirulli et al. (1999)
F12‐18	P12‐18	E1	c.33delC p.Lys12Argfs*34	Frameshift/deletion	Homozygous	Pirulli et al. (1999)
F19	P19‐20	E1	c.80G > A p.Gly27Glu	Missense	Homozygous	Current study
F20	P21	E7	c.766delC p.Gln256Serfs*17	Frameshift/deletion	Homozygous	Current study
F21	P22	E1	c.[65A > G];[1020A > G] p.[Asn22Ser];[Ile340Met]	Missense	Heterozygous	Williams et al. (2009), Pirulli et al. (1999)

^a^ Nucleotide changes are based on *AGXT* (NM_000030.2) reference sequences.

^b^ Protein changes are based on AGXT(NP_000021.1) reference sequences.

^c^ References refer to the first study to report the respective mutations.

### 3.3 Bioinformatics analysis of novel missense mutation (c.80G > a, p.Gly27Glu) on protein structure (Figure 2)

**FIGURE 2:**
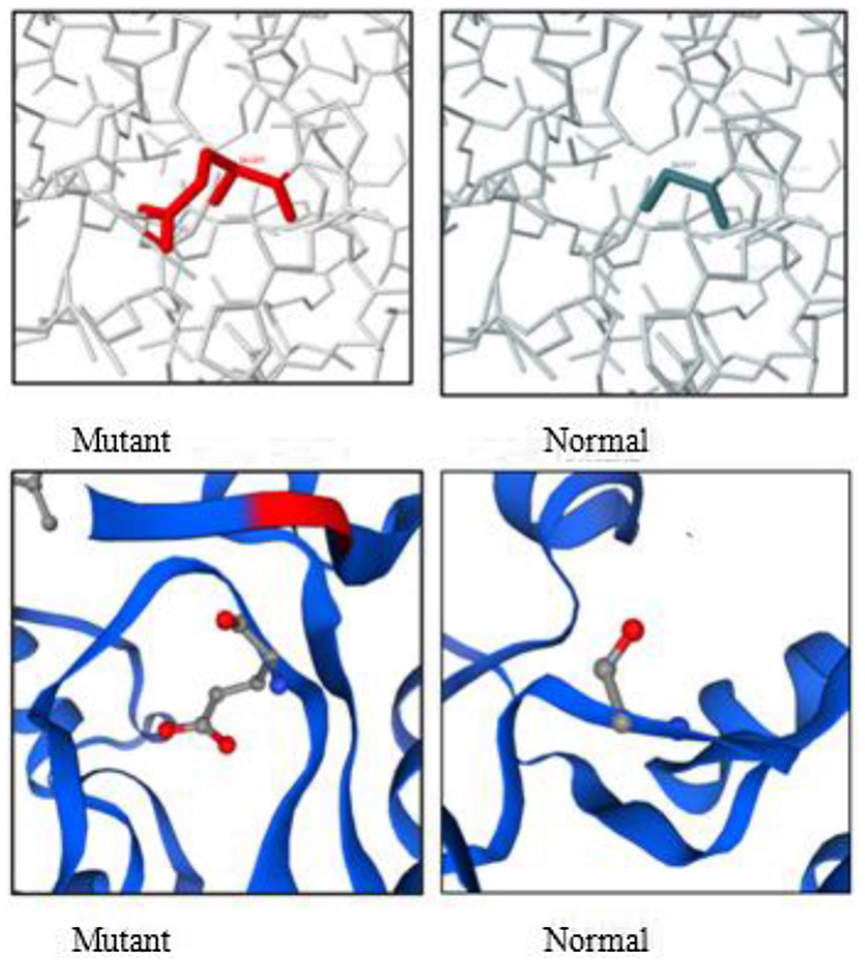
Bioinformatics analysis of a novel missense mutation (c.80G>A, p.Gly27Glu) on protein structure.

The effect of p.Gly27Glu on protein conformation was also analyzed using SAAMBE, missense3D, and PremPS. SAAMBE revealed that the Gly27Glu mutation leads to more destabilization of the protein with ΔΔG Prediction: 0.14 kcal/mol. Missense3D revealed that the Gly27Glu will damage the protein due to the following reasons:

- p.Gly27Glu substitution replaces a buried uncharged residue (GLY, RSA 1.1%) with a charged residue (GLU) (RSA 1.0%).
- P.Gly27Glu substitution triggers disallowed phi/psi alert. The phi/psi angles are in the favored region for wild‐type residue but the outlier region for mutant residue.
- PremPS predicted that the p.Gly27Glu mutation will result in a hydrophobic and ionic bonding that will affect the protein function.

## 4 DISCUSSION

Diagnosis of PH was based primarily on the clinical and biochemical findings. All patients in the current study exhibited the characteristic clinical and radiological manifestations of PH with variable age of onset. In Egyptian PH1 patients, urinary stones, nephrolithiasis, and ESRD were the clinical findings at the time of presentation similar to those described in previous reports (Boualla et al., 2015; He et al., 2019). The estimated prevalence varies from 1 to 3 in 1,000,000 in Europe and North America and 1–2% of pediatric ESRD population registries from the USA, UK, and Japan. In Egypt, we do not have an exact estimate of the prevalence of PH, however, the high consanguinity prevalence in a populous country such as Egypt might imply an expected high risk for inherited disorders, especially the autosomal recessive traits.

In this study, detailed clinical and molecular analyses of 22 patients with PH were performed. The results among our cohort revealed that all patients had *AGXT* mutations with no mutations detected in *GRHPR* and *HOGA1* genes. This coincides with previous estimates that PH1 is the most common form worldwide. In our cohort, the high prevalence of PH1 may be attributed to different factors: (i) later and milder presentation of PH2 and PH3, (ii) the inappropriate lack of awareness of this inherited disease, (iii) small sample size, and (iv) the presence of high homogeneity due to high consanguineous rate between Egyptian population.

All of our patients proved to carry homozygous or compound heterozygous variants in the *AGXT* gene comprising five missense and three frameshift mutations. Two of these variants were novel variants. The effect of c.80G > A(Gly27Glu) on protein structure was assessed using SAAMBE, missense 3D, and PremPS and is predicted to cause protein destabilization. Patients with c.80G > A (p.Gly27Glu) were presented with bilateral renal stones and nephrocalcinosis with no systemic oxalosis.

Most of the detected mutations in this study were found in exon 1 (5/8), which agreed with (Monico et al., 2007; Williams & Rumsby, 2007) that previously reported common mutations located in exons 1, 4, and 7 were responsible for 50% of PH1 patients. Regarding duplication C mutation p.Lys12Glnfs*156 in exon 1, it was accounted for the most commonly observed mutation among our cohort (7/22) in homozygous state and (4/22) in the compound heterozygous state with p.lle244Thr. This variant has been identified in several reports that resulted in a frameshift and premature termination and the formation of a truncated protein (Al Riyami et al., 2015). Patients who had this variant presented with recurrent bilateral urolithiasis, urinary tract infections, ESRD, and systemic oxalosis (Myocardial infiltrations, left ventricular hypertrophy, and bone pains). These clinical manifestations were similar to previous reports (He et al., 2019; Mbarek et al., 2011). The high frequency of c.33dupC in our study was in line with other reports from different populations with no apparent ethnic or geographic associations and its high frequency was attributed to the presence of multiple mutations in the region of eight cytosine repeat sequences (Al Riyami et al., 2015; He et al., 2019; Mbarek et al., 2011; Monico et al., 2007).

Compound heterozygous for p.Lys12Glnfs*156 and p.lle244Thr in addition to the coinheritance of both minor alleles (p.Pro11Leu and p.Ile340Met) were observed in four patients. Measurement of enzyme activity was done in two patients (2/4) and revealed poor AGT activity (2%). These patients presented with severe clinical manifestations in accordance with several studies that reported patients had compound heterozygous mutations in *AGXT* were complained about of severe symptoms such as recurrent urinary tract infections, urolithiasis, nephrocalcinosis with bone deformities, myocardial infiltration, and left ventricular hypertrophy (He et al., 2019, Mbarek et al., 2011). Kanoun et al. (2017) identified different variants of p.Lys12Glnfs*156, p.Ile340Met, p.lle244Thr, and p.Pro11Leu in two patients with severe manifestations suggesting that these mutations could destabilize the helical conformation of the peptide. Du et al. (2018) also identified p.Ile340Met as a risk variant that reduces 15.7% of AGT protein expression in PH1 patients. The mutational analysis in patient 22 showed the presence of reported variations p.Ile340Met and p.Asn22Ser in a compound heterozygous state with a mild phenotype. This patient had an AGT activity of <10%. These variants were the only detectable variants by the NGS panel for further functional analysis. Maybe there are deep intronic variants or gross deletions not detected by the panel.

There were controversial results in the identification of c.32C > T as a pathogenic variant. Nicolas et al. (2019) reported that this variant was not sufficient alone to cause protein dysfunction as the allelic frequency of p.Pro11Leu in the gnomAD database was ~0.15 (ranging from ~0.20 in Europeans to ~0.0004 in East Asians) in the general population. In in silico analysis, there is a conflict in the determination of pathogenicity of c.32C > T. In our patients, the presence of other two pathogenic variants with c.32C > T explained the disease phenotype and it is not responsible for the disease in our patients. Different studies proved its deleterious effect where it affects a highly conserved N‐terminal extended region of AGT protein, which is important in the process of dimerization and stabilization of the two protein subunits and it reduces the specific catalytic activity of purified recombinant AGT to about 30% of normal (Boualla et al., 2015; Kanoun et al., 2013, 2017).

Two frameshift deletion mutations were reported in this study; the first was p.Lys12Argfs*34 representing the second most common mutation in our cohort (8/22). All patients affected by this mutation were in a homozygous state while it was previously documented in homozygous and heterozygote states in PH1 patients from various ethnicities (Boualla et al., 2015; Williams & Rumsby, 2007). The second frameshift mutation was a novel mutation p.Gln256Serfs*17 in exon 7 found in one patient in the homozygous state. The patients presented with bilateral nephrolithiasis, nephrocalcinosis, ESRD, and systemic oxalosis. Our results also provide clinical evidence that the patients (19/22) with frameshift and compound heterozygote mutations had severe manifestations with systemic oxalosis and appearance of manifestations during the first year after birth, whereas patients (3/22) with missense mutations had less severe phenotypes. The median age at presentation for patients carrying frameshift and compound heterozygote mutations was (1 year and 2 months) while those carrying the missense mutations were older with median age (of 5 years and 7 months) which is in accordance with several previous reports (Du et al., 2018; Kanoun et al., 2017).

In conclusion, apart from adding novel mutations to the repertoire of known mutations in the disease, to the best of our knowledge, this is the first report of genetically characterized patients of PH from Egypt.

## AUTHORS’ CONTRIBUTION

Hoda Radwan, Mohamed A. Abdel Mawla designed the analyses, collected and interpreted the data, and wrote the manuscript. Hoda Radwan and Khalda Amr interpreted the molecular data and performed targeted sequencing. Mohamed A. Abdel Mawla, Fatina I. Fadel, and Doaa M. Salah performed the clinical evaluation of patients. All authors reviewed and approved the manuscript.

## CONFLICT OF INTEREST

All authors declare that they have no conflict of interest to disclose.

## ETHICS STATEMENT

The study was approved by ethical committee of National Research center. All procedures followed were in accordance with the Helsinki Declaration of 1964.
